# The Role of the Arabidopsis Exosome in siRNA–Independent Silencing of Heterochromatic Loci

**DOI:** 10.1371/journal.pgen.1003411

**Published:** 2013-03-28

**Authors:** Jun-Hye Shin, Hsiao-Lin V. Wang, Jinwon Lee, Brandon L. Dinwiddie, Dmitry A. Belostotsky, Julia A. Chekanova

**Affiliations:** School of Biological Sciences, University of Missouri–Kansas City, Kansas City, Missouri, United States of America; University of Massachusetts at Amherst, United States of America

## Abstract

The exosome functions throughout eukaryotic RNA metabolism and has a prominent role in gene silencing in yeast. In *Arabidopsis*, exosome regulates expression of a “hidden” transcriptome layer from centromeric, pericentromeric, and other heterochromatic loci that are also controlled by small (sm)RNA-based *de novo* DNA methylation (RdDM). However, the relationship between exosome and smRNAs in gene silencing in Arabidopsis remains unexplored. To investigate whether exosome interacts with RdDM, we profiled Arabidopsis smRNAs by deep sequencing in exosome and RdDM mutants and also analyzed RdDM-controlled loci. We found that exosome loss had a very minor effect on global smRNA populations, suggesting that, in contrast to fission yeast, in Arabidopsis the exosome does not control the spurious entry of RNAs into smRNA pathways. Exosome defects resulted in decreased histone H3K9 dimethylation at RdDM-controlled loci, without affecting smRNAs or DNA methylation. Exosome also exhibits a strong genetic interaction with RNA Pol V, but not Pol IV, and physically associates with transcripts produced from the scaffold RNAs generating region. We also show that two *Arabidopsis rrp6* homologues act in gene silencing. Our data suggest that Arabidopsis exosome may act in parallel with RdDM in gene silencing, by epigenetic effects on chromatin structure, not through siRNAs or DNA methylation.

## Author Summary

To maintain genomic stability and prevent expansion of invasive genomic sequences such as transposable elements (TEs), eukaryotes have evolved defensive mechanisms to control them. Here, we examine the role of the *Arabidopsis* exosome complex in such mechanisms. Evolutionarily conserved from archaea to humans, the exosome is a stable complex of RNase-like and RNA binding proteins that plays a central role in RNA metabolism in eukaryotes. Depletion of the exosome allows some repetitive sequences to escape from silencing. Most of these transcripts emanate from centromeric and pericentromeric chromosomal regions and other heterochromatic loci, and many derive from repetitive and transposable elements. In plants, TEs are targeted for *de novo* DNA methylation by smRNA–mediated pathways. However, we found that exosome depletion has only minor effects on smRNA populations that are acting in the main silencing mechanism in *Arabidopsis*, siRNAs–dependent DNA methylation RdDM. Instead, exosome depletion affects histone H3K9 dimethylation, an epigenetic mark that affects chromatin structure and thus alters transcription. Our data suggest that the exosome collaborates in gene silencing, likely acting in a parallel pathway to other mechanisms. We also propose that the *Arabidopsis* exosome may coordinate the transcriptional interplay of different RNA polymerases to modulate repression of some repetitive sequences.

## Introduction

High-throughput analyses have revealed that eukaryotic genomes are pervasively transcribed [1]–[4], and the majority of the transcriptional activity takes place outside of protein-coding genes, producing non-coding RNAs (ncRNAs) derived from genome regions once thought to be transcriptionally silent, including intergenic and heterochromatic regions [1]–[3], [5]. Pervasive transcription constitutes a risk for the cell, as it can be associated with expansion of TEs, loss of genomic stability and defects in gene expression. However, recent studies have also shown that ncRNAs themselves can have important regulatory functions, including the establishment and maintenance of the epigenetic architecture of eukaryotic genomes. In some cases, long ncRNAs serve directly as molecular scaffolds for recruiting chromatin modifiers [6], [7], whereas in other cases ncRNAs are processed by the RNAi machinery into short interfering siRNAs that guide DNA methylation and chromatin modifications to homologous regions of the genome [8], [9]. Thus, RNA-mediated heterochromatin formation requires an affected region to be transcribed for transcriptional silencing to occur. Many of the ncRNA transcripts earned the term “hidden” because they remain invisible unless RNA degradation is prevented, for example, by inactivation of the degradation machinery [1], [3], [4], [10]–[14], raising the important question of how these ncRNAs are regulated.

The exosome complex plays a central role in RNA metabolism in eukaryotes. Evolutionarily conserved from archaea to humans, the exosome is a stable complex of RNase-like and RNA binding proteins that catalyzes 3′ to 5′ processing and decay of various RNA substrates [15]. The current view of eukaryotic exosome structure is based mostly on studies done in yeast and human. The eukaryotic exosome has nuclear and cytoplasmic forms that share ten components. The key structural feature is a nine-subunit donut-shaped structure called the exosome ring. Six of the subunits, RNase PH domain-containing proteins Rrp41, Rrp42, Rrp43, Rrp45, Rrp46 and Mtr3, are organized into a hexameric ring, capped on one side by a trimer of subunits that contain S1 and KH RNA binding domains (Rrp40, Rrp4 and Csl4) [16], [17]. The 9-subunit ring structure has no catalytic activity in yeast and human, due to amino acid replacements that disable binding of RNA, phosphate ion, or catalysis [16], [17]. The exosome active sites are contributed by the tenth protein, Rrp44 (Dis3), which has endonucleolytic and exonucleolytic activities and considered to be the tenth subunit of the exosome core [18], [19]. In addition to Rrp44, the nuclear form of the eukaryotic exosome is associated with a second active 3′ to 5′ exonuclease, Rrp6 [20], [21]. Most functions of the exosome are dependent on cofactors. One of the notable complexes associated with the nuclear exosome is the Trf-Air-Mtr4 polyadenylation (TRAMP) complex endowed with a poly(A) polymerase activity that stimulates degradation [22]–[24]. The plant exosome might differ from yeast and human exosomes, as its ring subunit Atrrp41p appears to retain an active site and was also shown to have catalytic activity *in vitro* [1], [25]. Our previous genome-wide study using tiling microarrays to examine exosome targets in Arabidopsis revealed that a large number of exosome substrates correspond to ncRNAs originated from promoters, 5′UTRs, intergenic regions, repetitive elements and TEs [1]. Many of these ncRNAs derive from centromeric and pericentromeric regions and other heterochromatic loci known to give rise to smRNAs that participate in silencing of these loci [26]. In Arabidopsis, the main and most-studied pathway for transcriptional gene silencing of repetitive elements and transposons is the siRNA-based silencing mechanism known as RNA-dependent DNA methylation (RdDM) [9], [27]–[29]. The effects of exosome depletion on these ncRNAs and, potentially, on smRNAs are unlikely to be attributable to indirect effects of exosome depletion on the expression of RdDM pathway components, since no genes acting in siRNA biogenesis, siRNA-mediated transcriptional gene silencing (TGS), DNA methylation or demethylation, or histone H3K9 modifications were found to be affected in these lines [1].

RdDM induces *de novo* methylation of cytosines in all sequence contexts at the region of siRNA–DNA or siRNA-RNA sequence homology. This silencing pathway requires two plant-specific RNA polymerases, Pol IV and Pol V, specializing in transcriptional gene silencing (TGS) [28], although transcriptional activity of Arabidopsis Pol II was also reported to be involved in siRNA-directed gene silencing [30]. The mechanistic details of RNA-dependent silencing are not fully understood and also appear to vary from one genomic location to another, but the RdDM pathway likely consists of three main steps: (i) siRNA production from transcripts that are likely transcribed by RNA Pol IV [9], (ii) synthesis of non-coding RNAs that could serve as scaffolds by RNA Pol V and/or Pol II at some of the loci [30], [31], and (iii) assembly of AGO-siRNA effector complexes to recruit methylation machinery to complementary sequences [9]. In siRNA biogenesis, RNA Pol IV transcripts are made double-stranded by RNA-DEPENDENT RNA POLYMERASE 2 (RDR2), processed into 24 nt siRNA by DICER-LIKE 3 (DCL3), and then incorporated into ARGONAUTE (AGO4 and possibly AGO6) to form an AGO-siRNA complex [32]–[35]. The AGO-siRNA complex and other RdDM effectors [31], [35]–[37], assemble on scaffold RNA to form a guiding complex that recruits DNA methyltransferases and histone methyltransferases to direct the silencing of specific genomic loci through a mechanism that is not fully understood. Pol IV is thought to initiate RdDM pathway, whereas Pol V and AGO4-associated siRNAs function downstream from Pol IV to promote DNA methylation by recruiting the silencing complex to targeted loci. RNA Pol IV, Pol V and Pol II activities in RdDM are functionally diversified and coordinated; however, it is not yet clear how they are functionally integrated in heterochromatin silencing.

The model system in which siRNA-mediated silencing is the best understood mechanistically is fission yeast. In *S. pombe* RNA Pol II carries out the functions attributed to Pol IV and Pol V in plants, therefore, it generates both siRNA precursors and scaffold transcripts to which siRNAs bind at loci that are subject to siRNA-mediated silencing. Exosome defects in *S. pombe* were reported to result in the loss of transcriptional silencing from centromeric, silent mating type, and telomeric loci [38]–[40]. In *S. pombe*, in the absence of exosome-mediated degradation, abundant aberrant RNA species enter the RNAi pathway and interfere with heterochromatic silencing through competition for RNAi biogenesis machinery, resulting in the dramatic decrease in centromeric siRNAs [38]–[40]. Recently, it was also shown that exosome plays an important role in remodeling of facultative heterochromatin [41]. Earlier work in plants also suggested that aberrant RNAs could enter RNAi pathways unless they are degraded by the 5′ to 3′ pathway [42]. However, the role of the exosome complex in smRNA metabolism in Arabidopsis has not been examined. It is also not known whether the Arabidopsis exosome complex interacts with the RdDM silencing pathway.

To answer these questions we employed next-generation sequencing to profile populations of smRNAs in exosome-depleted plants, and in mutants of RdDM pathway genes. Unexpectedly, we found that loss of the exosome subunits had little effect on the global populations of smRNAs and had no affect on the level of DNA methylation in examined RdDM loci; rather, it resulted in a reduction of histone H3K9 dimethylation. We propose that the Arabidopsis exosome may coordinate the transcriptional interplay of RNA polymerases Pol II, Pol V and Pol IV, to achieve the appropriate level of transcriptional repression of heterochromatic loci.

## Results

### Exosome depletion does not affect smRNA profiles

Previously, we found that the majority of transcripts upregulated in RRP4 and RRP41 exosome depletion mutants originate from the promoters, repeats, intergenic, and siRNA generating regions [1]. Most of these regions harbor repeats and TEs that are known to be silenced by RdDM through siRNAs.

**Figure 1:**
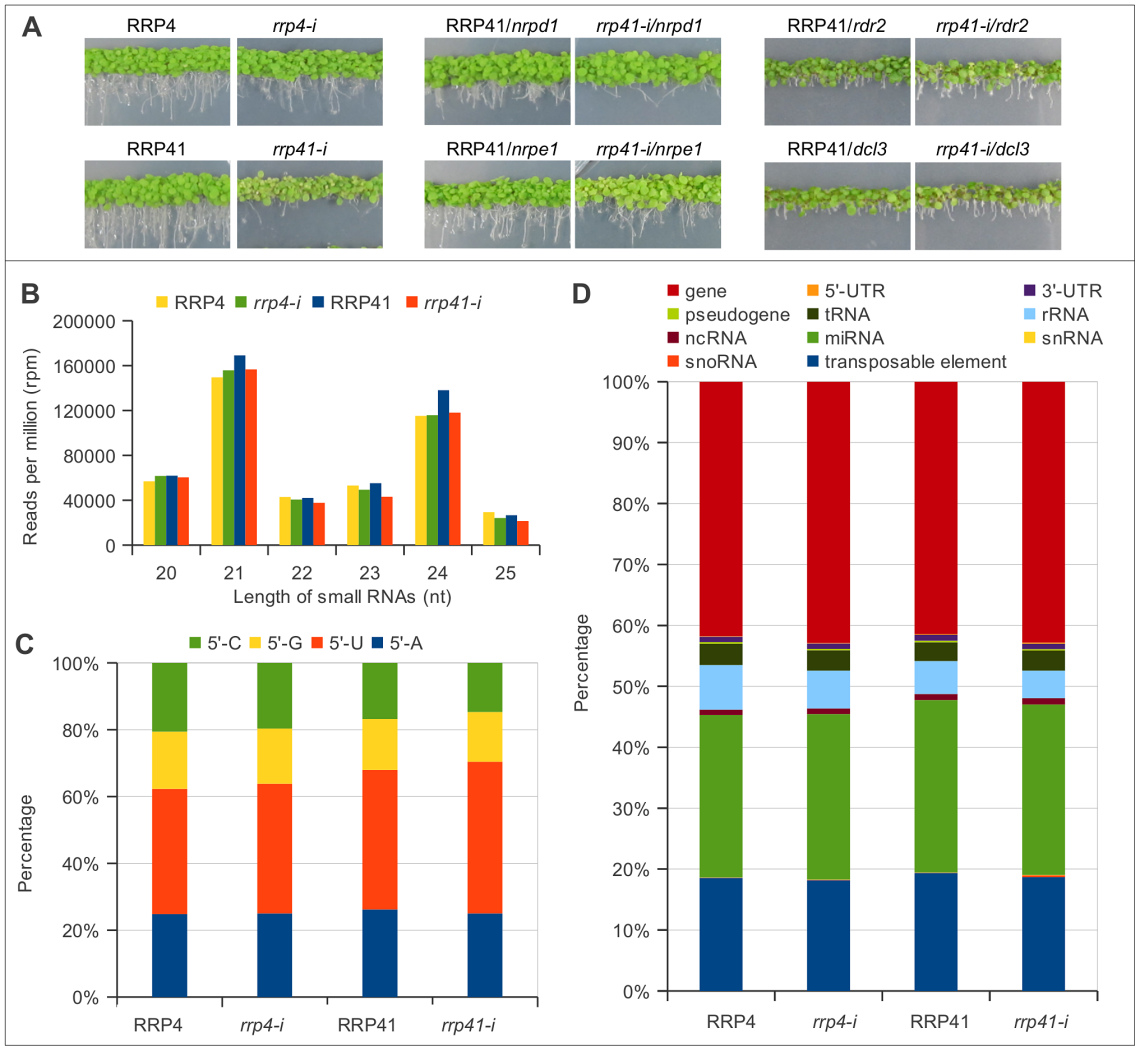
Characterization of up-regulated loci and smRNA populations upon depletion of exosome subunits RRP4 and RRP41. (A) Phenotypes of *rrp41 iRNAi/nrpd1, rrp41 iRNAi/nrpe1, rrp41 iRNAi/dcl3, rrp41 iRNAi/rdr2* double mutants. RRP4 and RRP41 correspond to the iRNAi lines grown without estradiol and *rrp4-i* and *rrp41-i* correspond to lines grown on estradiol-containing medium, to induce the RNAi-mediated knockdown of *RRP4* and *RRP41*, respectively. (B) 20–25 nt smRNAs sequences profiled by size in exosome depletion mutants *rrp4-i* and *rrp41-i*. (C) The relative frequency of each 5′ terminal nucleotide among populations 20–25 nt smRNAs in *rrp4-i* and *rrp41-i* mutants. (D) Genomic features of loci generating 20–25 nt small RNAs upon depletion of exosome subunits, according to TAIR9 annotation units.

Since microarray experiments allow estimation of only the length of affected regions, but not the exact length of affected transcripts, we set out to examine whether the exosome is involved in down regulation of these regions through regulating either quantity or quality of smRNAs. To characterize any changes in smRNA populations that occur in response to exosome depletion, we employed next-generation sequencing to deep sequence the smRNA populations in depletion mutants of exosome subunits RRP4 and RRP41. Null T-DNA insertion mutations in *RRP4* and *RRP41* are lethal; therefore, we used inducible RNA-interference (iRNAi) constructs to reduce RRP4 and RRP41. The seedlings of *RRP4* (*rrp4-i) or RRP41* (*rrp41-i*) transgenic plants grown on estradiol-containing medium to induce the RNAi constructs subsequently exhibit a growth arrest ([1], Figure 1A). We selected the earliest time-point of estradiol treatment corresponding to the accumulation of underprocessed 5.8S rRNA species (the hallmark of the exosome defect), but before growth retardation, to minimize changes in gene expression that did not result directly from exosome depletion [1]. Small RNA libraries for Illumina sequencing were generated from the seedlings of *rrp4-i* and *rrp41-i* iRNAi lines grown with and without estradiol (Table S1) and smRNAs between 15- and 32 nt in length were selected and mapped to the Arabidopsis genome (TAIR version 9).

We first examined the smRNAs from the *iRNAi* transgenes used for inactivation of *RRP4* or *RRP41* [1]. As expected, these silencing cassettes generate silencer sequences corresponding to *RRP4* or *RRP41* (mapping to AT1G03360 and AT3G61620 loci). Profiling silencer sequences by size and by first nucleotide revealed that the majority of the silencer sequences are 21, 22 and 24 nt and start with 5′U or 5′A (Figure S1), suggesting that they are preferentially loaded into Ago1, Ago2 and Ago4 complexes [43] to silence their target. Silencer sequences produced from *iRNAi* transgenes were filtered out and libraries without silencer reads were termed FLR, for filtered reads (Table S1). Each library was normalized either to the total number of mapped non-redundant reads or to the total number of non-redundant filtered reads (FLR), multiplied by 10^6^ (RPM, reads per million). Both methods of normalization produced similar results; therefore, only data normalized using filtered reads (FLR) are presented graphically in this study.

We then classified smRNAs based on their size, the nature of their first nucleotide, and their genomic features. The majority of functional smRNAs in *A. thaliana* range from 21 to 24 nt. Our libraries were constructed using 15–32 nt smRNAs; therefore, we were able to detect any effect exosome depletion might have on smRNA metabolism. We found that exosome defect does not lead to changes in smRNAs in the 15–19 nt and 26–32 nt categories (data not shown). Importantly, the group of 20–25 nt smRNAs, which contains the majority of functional smRNAs, was present in similar proportions, although with minor variations, relative to the number of total reads in the libraries of both of exosome depletion mutants and in their corresponding non-induced lines, and constituted about half of total smRNAs mapped to the genome (Table S1, Figure 1B). Therefore, for simplicity we graphed only data corresponding either to 20–25 nt smRNAs, or to smRNAs of one specific length.

In addition, the depletion of either RRP4 or RRP41, which are both essential for exosome function, with slight variations, had no effect on the smRNA size distribution (Figure 1B) or the frequencies of their first nucleotide (Figure 1C). All together, these results suggest that defects in exosome function do not lead to accumulation of un-degraded smRNA fragments or to any changes in the cleavage bias of Dicer proteins. Also, exosome depletion did not change proportions of smRNAs mapped to different classes of RNAs, such as mRNAs, tRNAs, rRNAs, and snoRNAs (Figure 1D). Therefore, unlike the situation in *S. pombe*, where exosome acts as a negative regulator of siRNA biogenesis, Arabidopsis exosome does not act to prevent spurious RNAs from entering RNAi pathway.

### Exosome depletion does not affect populations of smRNAs corresponding to repeats and transposable elements

**Figure 2:**
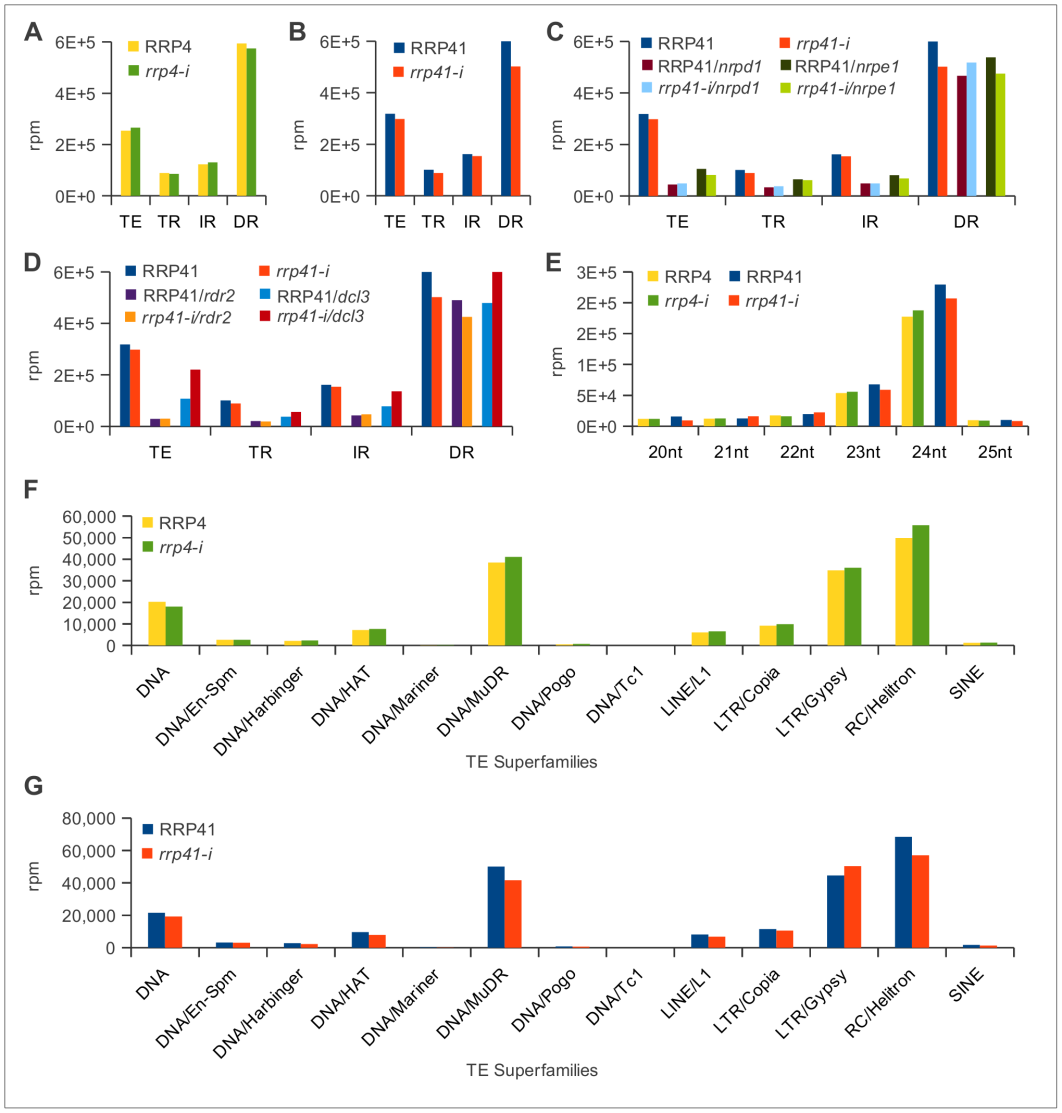
Characterization of 20–25 nt smRNAs corresponding to transposons and repeats in exosome and RdDM mutants. (TE = transposable element; TR = Tandem repeat; IR = Inverted repeat; DR = Dispersed repeat) (A) Results of depletion of exosome *rrp4* subunit. (B) Results of depletion of *rrp41* exosome subunit. (C) Results of depletion of *rrp41* in *nrpd1* and *nrpe1* genetic backgrounds. (D) Results of depletion of *rrp41* in *dcl3* and *rdr2* mutants. (E) Characterization of smRNAs mapped to repeats and transposable elements in *rrp4-i* and *rrp41-i* libraries profiled based on the reads length (F, G). Classification of 24 nt smRNAs corresponding to the different superfamilies of TEs in *rrp4-i* (F) and *rrp41-i* mutants (G) [76].

In Arabidopsis, repeats and TEs are silenced by siRNAs through RdDM; therefore, we examined the effect of exosome loss on 20–25 nt smRNAs corresponding specifically to TEs and repeats. Surprisingly, we saw no changes in the groups of smRNAs mapped to tandem repeats (TR), inverted repeats (IR), dispersed repeats (DR) or the group of TEs in both exosome mutants (Figure 2A and 2B). The diverse heterochromatic siRNAs participating in TE silencing are mostly 24-mers and are Pol IV- and/or Pol V-dependent [9]. Most siRNA production relies on Pol IV, but there are also Pol V-dependent and Pol IV-independent siRNA-generating loci [44], [45]. Therefore, to examine whether the exosome complex functionally overlaps with the components of the RdDM pathway, we constructed lines containing *rrp4-i* or *rrp41-i* iRNAi and mutations affecting Pol IV, Pol V, RDR2 and DCL3, which are *nrpd1*, *nrpe1*, *dcl3* and *rdr2* respectively (allele numbers provided in Methods). This approach also allowed us to confirm that smRNAs observed in exosome depletion lines are siRNAs produced by components of the RdDM pathway and not short RNA degradation products accumulated in the absence of functional exoribonucleolytic complex.

Pol IV, Pol V, RDR2 and DCL3 are not essential for viability [27], [29], [46]. Combining mutations in *nrpd1*, *nrpe1*, *dcl3* and *rdr2* with *rrp41-i* iRNAi knock-down line did not exacerbate the phenotypes of single exosome depletion mutants (Figure 1A).

**Figure 3:**
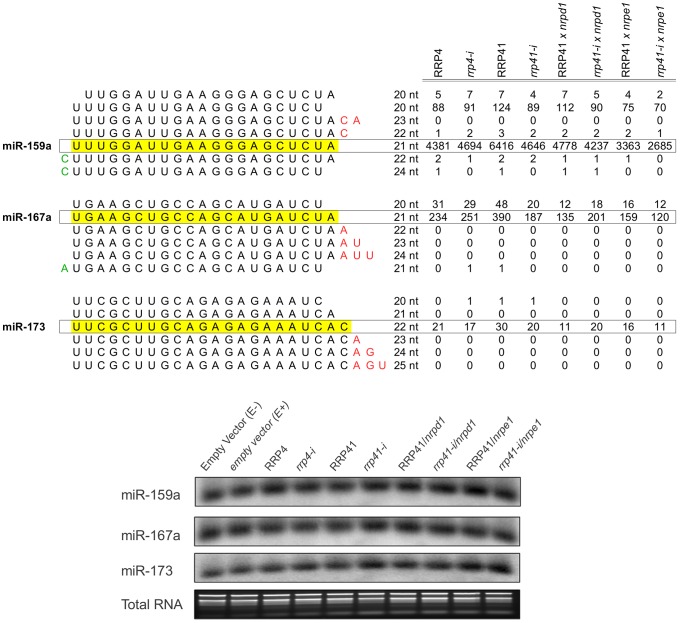
Expression of miRNAs in exosome mutants. miRNA families, miR-159a, miR-167a, miR-173 and variations in sequence length in each family. smRNAs mapped to matching mature miR-159, miR-167miR-173, and miR-167 sequences [94](miRBase release 18) were plotted versus the sum of their normalized reads per million (rpm) from smRNA libraries constructed from RRP4, *rrp4-i*, RRP41, *rrp41-i*, RRP41*/nrpd1, rrp41 iRNAi/nrpd1*, RRP4 *iRNAi/nrpe1 and rrp41 iRNAi/nrpd1* mutants. Detection of miRNAs by Northern Blot analysis demonstrates that mature miRNA levels are not affected by exosome depletion, and confirms the results of bioinformatic analysis. Total RNA stained with ethidium bromide was used as a loading control.

We next analyzed the smRNAs corresponding to repeats and TEs produced in the *rrp41*/*nrpd1* and *rrp41*/*nrpe1* double mutants (Figure 2C) and the *rrp41*/*rdr2* and *rrp41*/*dcl3* double mutants (Figure 2D). Similar to previous reports, we observed a significant reduction in the amount of smRNAs corresponding to TEs, TRs and IRs in *nrpd1*, *nrpe1*, *rdr2*, and *dcl3* mutants [27], [44], [47], [48]. Depletion of the exosome in *nrpd1*, *nrpe1* and *rdr2* mutants had no effect on the amount of TE and repeat-associated smRNAs produced in these mutants (Table S2, Figure 2C and 2D). Depletion of *rrp41* in *dcl3* led to a minor restoration of this defect in all groups of repeats and TEs. In the absence of *dcl3*, other *Arabidopsis* Dicer proteins are known to process *dcl3* substrates [49]; therefore this minor restoration most likely resulted from compensatory effects of other DICER proteins (Table S2, Figure 2D). Profiling repeat- and transposable element-generated smRNAs by their size confirmed that the exosome defect did not affect the group of 20–25 nt smRNAs even in Pol IV, Pol V, RDR2 and DCL3 deficient genetic backgrounds. Typically, siRNAs participating in RdDM are 24 nt long; therefore we profiled smRNAs mapping to transposable elements by length, but observed no change in abundance of 24 nt smRNAs (Figure 2E). Further analysis of the 24 nt smRNAs mapped specifically to the different transposable element superfamilies led to the same conclusion (Figure 2F and 2G). We therefore concluded that there are no significant changes in the populations of siRNAs corresponding to repeats and TE superfamilies in exosome depletion mutants. We also did not observe any significant differences in amounts of mature 21-mer miRNAs. The results of our sequencing analysis were confirmed by Northern blot analysis (Table S3, Figure 3, Figure S2). Together, these data suggest that the Arabidopsis exosome complex is not involved in siRNA metabolism on a global scale. Nevertheless, we can not exclude the possibility that exosome might control a small number of smRNA precursor transcripts at a few specific loci that would have been missed in our experiments and with the data processing approach we took while dissecting differences on genomic level.

### The exosome controls expression of ncRNAs in RdDM-regulated loci

**Figure 4:**
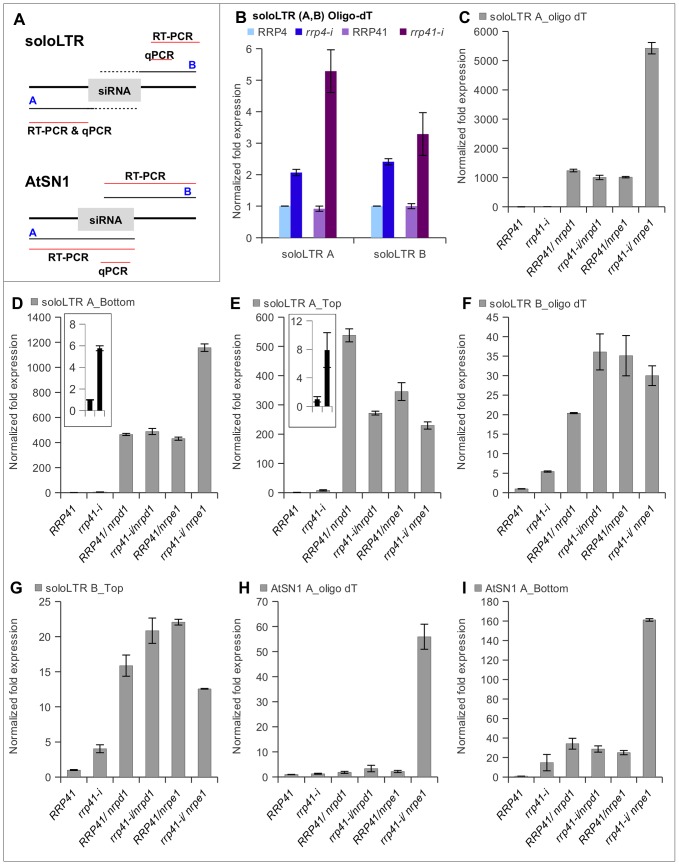
Effect of exosome subunits depletion on expression of ncRNA transcripts from RdDM-regulated loci. (A) Diagrams of solo LTR and AtSN1 loci, based on analysis of transcription units by Wierzbicki et al. (2008). Region A corresponds to the siRNA-producing region of solo LTR, region B corresponds to the adjacent to solo LTR region that produces scaffold RNA, and red lines mark regions amplified by RT-PCR and qPCR. The dotted line corresponds to the region of scaffold RNAs hypothesized to be complementary to the siRNAs produced from region A. (B) Depletion of exosome subunits RRP4 and RRP41 leads to an increase in noncoding transcripts generated from siRNA-producing region A and scaffold RNA-producing region B of solo LTR. RT was primed with oligo(dT). (C, D, E) Expression of region A of solo LTR in exosome depleted plants and various mutants. (C) Combining depletion of RRP41 with mutation in Pol V leads to a synergistic increase in accumulation of transcripts from region A. Strand-specific RT-PCR analysis revealed that both top and bottom transcripts of region A are affected by depletion of RRP41 subunit, but only bottom transcript is synergistically affected in *rrp41-i/nrpe1* double mutants (D), while the amount of top transcript is decreased in both *rrp41-i/nrpd1* and *rrp41-i/nrpe1* double mutants (E). (F, G) Expression of region B of solo LTR in exosome depleted plants and various mutants. (F) Depletion of *rrp41* leads to increased amounts of transcript produced from both strands of region B. (G) RT-PCR analysis of solo LTR top transcript. (H, I) Depletion of exosome subunit RRP41 leads to increase in ncRNA transcripts generated from the AtSN1 region. (H) Combining depletion of RRP41 with mutation in Pol V leads to synergistic increase in accumulation of polyadenylated transcript from region A. (I) Amount of the region A bottom strand of AtSN1 is synergistically increased in *rrp41-i/nrpe1* double mutants.

To further investigate whether the exosome participates in gene silencing and interacts with the RdDM pathway, we examined the transcription patterns of several specific loci regulated through RdDM. solo LTR and AtSN1 are the heterochromatic loci for which the role of RdDM players in their silencing and interactions between them are best-understood [30], [31], [50]–[52]. Transcriptional silencing of solo LTR and AtSN1 heterochromatic loci are dependent on Pol IV and Pol V [30], [31], [50]–[52]. Based on previous studies, both solo LTR and AtSN1 loci can be subdivided into region A and an adjacent region B [30], [31]. Region A represents the siRNA-generating region likely transcribed by Pol IV, and region B gives rise to the ncRNAs that are proposed to serve as a scaffold for recruiting siRNA-mediated complexes that form heterochromatin (Figure 4A). Pol V was proposed to produce transcripts which serve as the scaffolds [31], although in case of solo LTR, Pol II was also shown to be involved [30].

We then used real-time RT–PCR to examine the levels of transcript produced from region A, as a measure of the silencing status of each locus. We found that exosome defects resulted in accumulation of polyadenylated ncRNA produced from both regions A and B of solo LTR (Figure 4B). We then compared the amplitudes of the region A derepression in the *rrp41*, with *rrp41 iRNAi/nrpd1* and *rrp41 iRNAi/nrpe1* double mutants relative to the respective single mutants. As previously reported by others [30], [31], we observed solo LTR to be significantly derepressed in Pol IV and Pol V single mutants (Figure 4C and 4F). Interestingly, only the combination of exosome defect with mutation of Pol V, but not with mutation of Pol IV, resulted in the synergistic increase of region A transcript (Figure 4C). Reverse transcription with oligo dT primers does not discriminate between transcripts originating from either DNA strand; thus an elevated level of polyadenylated transcript could result from transcription from either one of the DNA strands. Therefore, to find out which of the transcripts increased in abundance, we carried out strand-specific RT-PCR for the A and B regions.

Following standard nomenclature, the top transcript (also called top strand RNA) corresponds to the transcript identical to the sequence of the DNA top strand (and therefore produced from the bottom DNA strand), and the bottom transcript is identical to the sequence of DNA bottom strand. The scaffold RNAs were reported to correspond to region B top strand [30], [31].

Similar to previous results [30], [31], we observed region A top and bottom transcripts to be significantly derepressed in Pol IV and Pol V single mutants (Figure 4D and 4E), and depletion of RRP41 lead to increased accumulation of the region A top and bottom transcripts (inserts in Figure 4D and 4E). Interestingly, we found that the bottom transcript was synergistically derepressed in *rrp41 iRNAi/nrpe1* double mutants relative to *nrpe1 and rrp41 iRNAi* single mutants, while no change was observed in *rrp41 iRNAi/nrpd1* double mutants (Figure 4D). Despite the fact that the exosome defect equally affected the levels of both top and bottom region A transcripts, combining the exosome defect with either Pol IV or Pol V mutants had no additive or synergistic effect on the level of region A top transcript. Surprisingly, the level of expression of region A top transcript was even somewhat decreased in *rrp41 iRNAi/nrpd1* and *rrp41 iRNAi/nrpe1*, compared to *nrpd1* and *nrpe1* single mutants, opposite to the pattern we observed for the bottom strand (Figure 4E). Production of scaffold transcripts is central in silencing of the locus and it was reported that even in the presence of functional Pol IV and siRNAs, silencing of solo LTR fails when scaffold RNAs are not produced [30], [31].

We therefore examined the scaffold-producing region B and found that the exosome also affects the amount of region B top transcript, but there is no synergistic increase of this transcript in *rrp41 iRNAi/nrpe1* double mutants (Figure 4F and 4G). When we examined AtSN1, we observed a very similar synergistic increase in the level of the siRNA-producing region A of bottom strand transcript of AtSN1 in *rrp41 iRNAi/nrpe1* mutants (Figure 4H and 4I).

Together, these results suggest that the exosome participates in controlling the amount of top transcripts emanating from the scaffold-producing region B of solo LTR, and thus may contribute to the repression of region A through regulating the level of region B transcripts.

### RRP41 depletion does not affect *de novo* DNA methylation in solo LTR and AtSN1 loci

**Figure 5:**
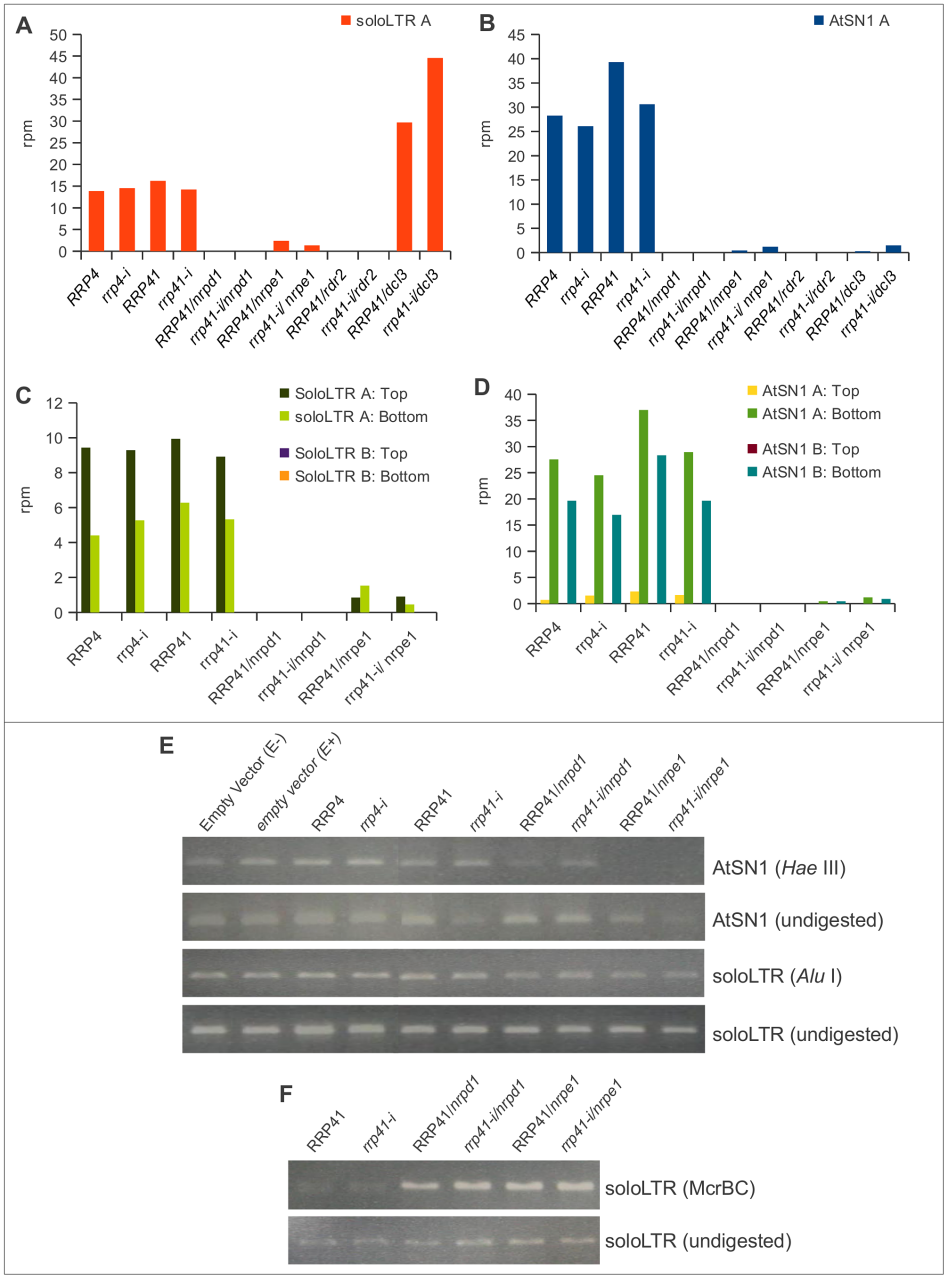
smRNA accumulation and DNA methylation in solo LTR and AtSN1 loci is unaltered upon exosome depletion. (A, B) 20–25 nt smRNAs produced from region A of solo LTR (A) and region A of AtSN1 (B) in *rrp4-i, rrp41-i* exosome depletion lines and RdDM mutants. All locus-specific datasets of 20–25 nt smRNAs are plotted versus the sum of their normalized reads per million (rpm). (C, D) Strand-specific analysis of smRNAs generated at regions A and B of AtSN1 (C) and solo LTR (D) loci in different mutants. (E, F) DNA methylation analysis of AtSN1 and solo LTR loci by digestion of purified DNA with the methylation-sensitive endonucleases HaeIII for AtSN1 (E), AluI for solo LTR (E), and McrBC for solo LTR (D), followed by PCR.

The solo LTR, AtSN1 and IGN5 loci are silenced primarily by RdDM, through siRNA mediated *de novo* methylation of DNA [9], [30], [31]. We reasoned that if the exosome is involved in controlling the amount of RNA expressed from these loci in a siRNA-dependent manner, then the exosome defect might affect the amount of siRNAs generated from these regions. To address this question, we first compared solo LTR and AtSN1-specific smRNAs. We found that production of smRNAs from the siRNA-generating A regions was not altered in *rrp4-i* or *rrp41-i* mutants relative to WT (Figure 5A and 5B), similar to the results of the global smRNA analysis we described above. The increased amount of smRNAs observed in *dcl3* mutants is because in the absence of DCL3, the other Dicer proteins process DCL3 substrates [49]. In order to make sure that the smRNAs produced from one strand of region A are not masking the smRNAs produced from the opposite strand in exosome depletion mutants, we also analyzed these smRNA populations in a strand-specific manner. However, the patterns of strand-specific siRNAs were very similar to the patterns we observed previously and siRNAs were not affected by exosome depletion (Figure 5C and 5D). We examined an additional region controlled by RdDM, the IGN5 locus [31], and found that IGN5-specific smRNAs are also not affected in exosome mutants, similar to solo LTR and AtSN1 loci (Figure S3C). This implies that the increase in accumulation of transcripts in exosome-depleted plants was not a result of siRNA defect. To verify this directly, we examined the patterns of DNA methylation in these regions by using methylation sensitive restriction enzymes (Figure 5E). The DNA of the solo LTR region was examined by two different assays (Figure 5E and 5F). We found that, consistent with the results of the region-specific siRNA analysis, *de novo* DNA methylation was not changed in *rrp41-i* plants (Figure 5A–5D). Taken together, these results indicate that an increase in transcript accumulation is not caused by the loss of *de novo* methylation and the region is still silenced by RdDM. It also suggests that in the examined loci, the exosome complex functions independently of RdDM. Interestingly, the increased amount of transcripts accumulated in these regions does not contribute to increased smRNA amounts in the exosome-depleted plants. This was observed regardless of whether these transcripts originated from siRNA-generating regions, or adjacent regions. Indeed, even several thousand-fold upregulation of region A transcript in *iRNAi/nrpe1* mutants (Figure 4B, 4C, 4G and 4H) does not produce any increase in the amount of siRNAs (Figure 5A–5D).

### H3K9me2 levels are affected in exosome-depleted plants

DNA methylation and histone modification are two major epigenetic marks regulating gene expression and chromatin state in plants. Monomethylated histone H3 lysine 27 (H3K27me1) and dimethylated histone H3 lysine 9 (H3K9me2) are hallmarks of heterochromatin, and silencing of solo LTR, AtSN1 and IGN5 loci also involves histone modifications [30], [31]. Although *de novo* methylation does not directly affect the level of H3K9me2, it does affect the level of H3K27me1 [31], suggesting that in addition to histone modification pathways, which are dependent on RdDM, other, RdDM-independent, pathways also contribute to transcriptional silencing of these regions. We therefore used chromatin immunoprecipitation (ChIP) to examine whether the exosome is involved in regulation of histone modifications in these loci.

**Figure 6:**
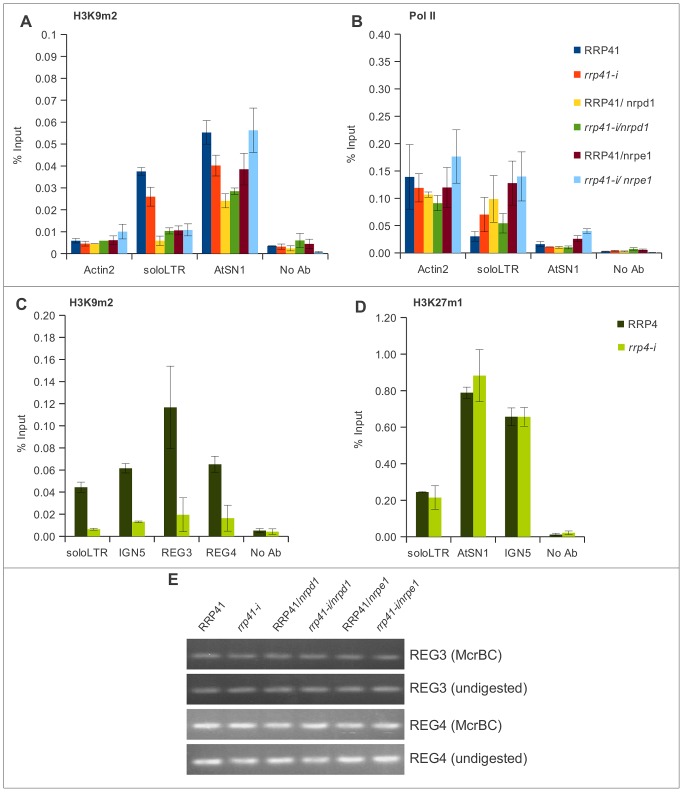
The effect of the exosome subunits depletion on the levels of H3K9me2 in different loci. (A, B) The levels of H3K9me2, and Pol II occupancy at solo LTR and AtSN1 examined by ChIP in RRP41, *rrp41-i*, RRP41/*nrpd1*, *rrp41-i/nrpd1*, RRP41/*nrpe1*, and *nrpd1/nrpe* mutants using antibodies against H3K9me2 (A), and RNA Pol II (B), respectively. (C) Effect of *RRP4* depletion on levels of H3K9me2 examined by ChIP at solo LTR, IGN5, REG 3, and REG 4 (C), and on levels of H3K27me1 at solo LTR, AtSN1, and IGN5 loci (D). No Ab, ChIP with no antibody, is used as a negative control. (E) Analysis of DNA methylation in REG 3 and REG 4 regions by McrBC treatment in RRP41, *rrp41-i*, RRP41/*nrpd1*, *rrp41-i/nrpd1*, RRP41/*nrpe1*, and *nrpd1/nrpe1* mutant plants. REG 3 and REG 4 are not methylated in wild type plants and no changes were observed in mutants. The error bars in ChIP experiments represent the standard error of the mean (SE) and correspond to the difference between 2 biological replicates.

Similar to the results reported by others [30], [31], we observed a significant decrease in the level of H3K9me2 in the solo LTR locus in *nrpd1* and *nrpe1* mutants, which affect Pol IV and Pol V, respectively. We found that RRP41 depletion also led to a decrease in H3K9me2 but less than observed in *nrpd1* and *nrpe1* mutants (Figure 6A). The decrease in level of this repressive histone modification also correlated with a mild increase in RNA Pol II occupancy in the solo LTR region, as would be expected with a release of transcriptional block (Figure 6B). The *rrp41 iRNAi/nrpe1* double mutant did not exhibit any additive or synergistic effect on the loss of H3K9me2 relative to respective single mutants.

When we examined AtSN1, we found that the level of H3K9me2 was mildly decreased in all mutants tested (Figure 6A). For AtSN1, it was previously suggested that RNA Pol III is the main RNA polymerase transcribing the region when the region is in a derepressed state [31], although RNA Pol II was also reported to be associated with this region [30]. We found that RNA Pol II occupancy in AtSN1 was very low but it increased significantly in *rrp41 iRNAi/nrpe1* double mutants (Figure 6B), in accordance with the synergistic increase of the transcript level we observed (Figure 4H and 4I).

Depletion of another exosome subunit, RRP4, caused a similar loss of H3K9me2 at solo LTR and AtSN1 loci (Figure 6C). We then chose several additional regions, termed REG3 and REG4 (Figure S3A), that are mildly upregulated in exosome mutants according to our previous microarray analysis [1], and examined them using ChIP. REG3 harbors a MuDR transposon, and REG4 is situated in a tandem repeat area. Neither of these loci produces smRNAs or is controlled by DNA methylation (Figure 6E and data not shown). We found that the H3K9me2 in these loci was similarly affected by exosome depletion (Figure 6C), while the level of H3K27 methylation in these regions didn't show any difference (Figure 6D). These results suggest that the exosome may participate in maintaining chromatin structure in these regions as well, and does so by specifically affecting the level of H3K9me2 in addition to controlling the level of transcripts.

### Exosome associates with transcripts produced from a scaffold-generating area adjacent to solo LTR

**Figure 7:**
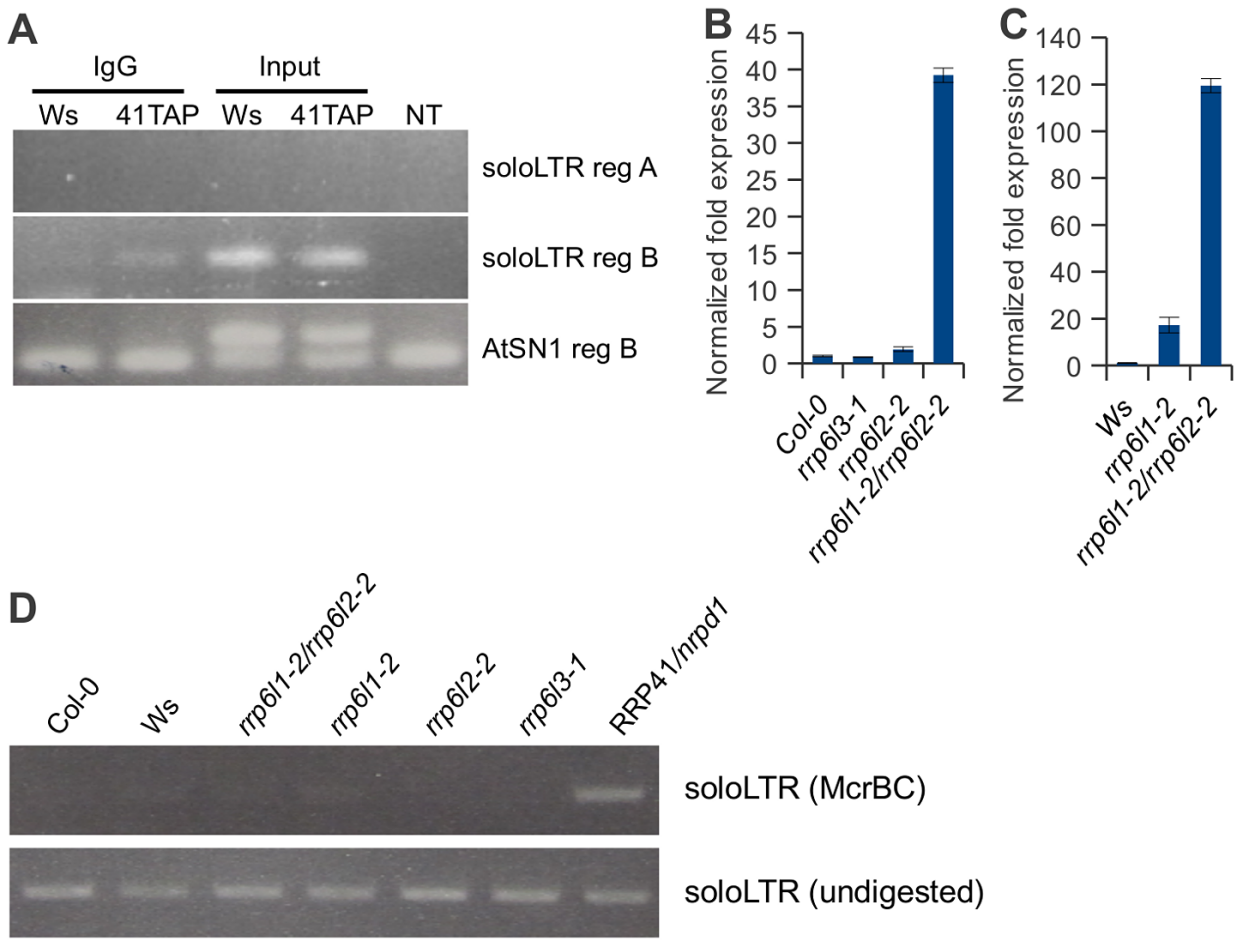
Exosome associates with transcripts produced from the region adjacent to the solo LTR scaffold-generating area. (A). RT-PCR of RNA-immunoprecipitation using plants carrying a functional RRP41-TAP transgene and empty-TAP transgene to examine the association of exosome with noncoding transcripts produced at siRNA and scaffold RNA producing loci. Region B transcripts were co-precipitated with exosome, while no region A transcripts were detected in immunoprecipitates. Transgenic plants are in Ws ecotype. (B, C) Two homologous *rrp6* catalytic subunits of exosome are involved in controlling the amount of ncRNA emanating from the solo LTR locus. (C) Expression pattern of region A of solo LTR locus in *rrp6l3-1*, *rrp6l2-2, 6l1-2*, and the double mutant *rrp6l1-2*/*rrp6l2-2* compared relative to the RNA expression in Col-0 ecotype wild-type (B), compared relative to the RNA expression in Ws ecotype wild-type (C). (D) Analysis of DNA methylation in solo LTR by McrBC treatment in Col-0, Ws, *rrp6l1-2*, *rrp6l2-2*, double mutant *rrp6l1-2*/*rrp6l2-2*, *rrp6l3-1*mutant plants. RRP41/*nrpd1* mutant DNA is used as a control.

We then examined exosome association with ncRNA loci. Detection of some protein–nascent mRNA interactions by ChIP were reported previously for proteins working on RNA, but the results of our attempts to localize tagged exosome subunits at solo LTR locus have proven inconclusive. Transcripts from region A are normally below the level of detection in wild-type plants, but transcription from the region B adjacent to solo LTR has been previously documented in wild-type plants [1], [30], [31]. In order to confirm that the exosome directly associates with these transcripts, we conducted RNA immunoprecipitation (RIP) using plants carrying a transgene expressing RRP41-TAP, and examined the ncRNAs associated with the exosome by RT-PCR. No region A transcripts were detected in immunoprecipitates, but we found that region B transcripts were co-precipitated with exosome (Figure 7A). These data suggest that in wild-type plants, exosome physically associates with polyadenylated transcripts produced from region B of solo LTR.

In contrast to solo LTR, we did not detect a physical association of exosome with AtSN1 region B transcript (Figure 7A). This implies that exosome depletion may not directly affect the silencing of AtSN1. However, we observed that exosome depletion resulted in accumulation of transcript in the AtSN1 locus and we detected a synergistic derepression of the locus in *rrp41/nrpe1* mutants, similar to solo LTR locus (Figure 4H and 4I). Most likely the regulation of AtSN1 is more complex because an additional RNA polymerase, RNA Pol III, is involved. AtSN1 is transcribed mostly by RNA Pol III [31], [53], suggesting that the double deficiency in exosome and Pol V may increase both Pol II and Pol III access to the locus. We also observed the increased Pol II association with AtSN1 in rrp41/nrpe1 mutants by ChIP assay using anti-Pol II (Figure 6B), which is consistent with the results of qRT-PCR. Therefore, it is also possible that the loss of exosome function may lead to the alteration of chromatin structure in regions adjacent to AtSN1 and thus affect the stability of silencing in AtSN1 indirectly. Nevertheless, these results are similar to the interplay between exosome and Pol V observed for solo LTR.

### RRP6 is involved in controlling levels of ncRNA from the solo LTR locus

The 9-subunit exosome complex is catalytically inactive in yeast and human. Instead, active sites are contributed by Rrp44 (Dis3) and by the subunit Rrp6, which is substoichiometric, nuclear-specific, and not essential for viability. Degradation of *S. cerevisiae* nuclear ncRNAs depends on polyadenylation by the TRAMP complex and involves Rrp6, the subunit that is also responsible for elimination of heterochromatic RNAs in *S. pombe* [20], [22]–[24], [39]–[41]. In Arabidopsis there are three RRP6-like proteins – nuclear localized RRP6L1 and RRP6L2, and cytoplasmic RRP6L3; these were suggested to be functional homologues of RRP6 [54]. None of the RRP6-like proteins co-purified with the exosome complex in our proteomic studies [1], but may have been underrepresented in our preparations. In addition, RRP6L2 was later shown to have at least some commonalities with core exosome substrates [54]. We therefore examined whether the Arabidopsis RRP6-like proteins control the amount of ncRNA at the solo LTR locus. To determine this, we used T-DNA insertion alleles in *RRP6L1*, *RRP6L2* and *RRP6L3*. We isolated the *rrp6l1-2* allele from the University of Wisconsin BASTA population (Ws ecotype), and the alleles of the *rrp6l2-2* and *rrp6l3-1* are SALK alleles (Col-0 ecotype). To control for effects of ecotype, we compared the amount of region A transcript in *rrp6l3-1*, *rrp6l2-2*, *rrp6l1-2/rrp6l2-2* mutants to Col-0 wild type plants, and *rrp6l1-2*, *rrp6l1-2/rrp6l2-2* mutants to Ws ecotype plants (Figure 7B and 7C).

We found that, similar to depletion of the core subunits RRP4 and RRP41, *rrp6l1-2* and *rrp6l2-2* mutants exhibited increased accumulation of transcripts produced from region A. As would be expected based on cytoplasmic localization of RRP6L3 protein, no effect was observed in *rrp6l3-1* mutants. To our surprise, we observed a dramatic derepression of region A in *rrp6l1-2/rrp6l2-2* double mutants, suggesting that both RRP6L1 and RRP6L2 proteins are involved in the silencing of this region and might have a redundant function in this process.

We also examined the status of solo LTR DNA methylation in *rrp6l1-2, rrp6l2-2, and rrp6l1-2/rrp6l2-*2 double mutants. We found that methylation was not affected in these mutants regardless of the extent of derepression of the region (Figure 7D), consistent with the results obtained using *rrp4-i* and *rrp41-i* depletion mutants. Taken together, these results indicate that the observed increase in transcript accumulation is not caused by the loss of *de novo* methylation and the region is still methylated by RdDM. This further confirms that the exosome complex functions independently of the RdDM pathway.

## Discussion

### The exosome and smRNA metabolism

The exosome functions in virtually all aspects of RNA metabolism and it appears to also have a prominent role in transcriptional gene silencing in different species [1], [10], [38]–[41], [55]–[59]. This study examined the role of the exosome complex in metabolism of smRNAs and explored the possible relationship between the exosome and the RdDM pathway in gene silencing in Arabidopsis.

Our results showed that exosome-mediated silencing did not produce global changes in smRNA profiles, nor in DNA methylation at specific loci. However, we did find effects on histone methylation, indicating that the exosome may regulate chromatin structure, thereby playing an important role in maintenance of gene silencing on a much broader scale than the RdDM pathway. It is clear from our results using suppression of key exosome components that plants have an exosome-dependent pathway that relies on ncRNAs to target heterochromatin.

Our finding that the increase in ncRNA transcribed from heterochromatic loci in exosome-depleted plants did not lead to an increase in levels of smRNA indicates that exosome function in Arabidopsis differs from that in fission yeast. In fission yeast, exosome defects have a dramatic effect on siRNAs leading to redistribution of the spectrum of Ago1-associated siRNAs, from mostly repeat-associated to those derived predominantly from exosome substrates such as rRNA and tRNA [39], indicative of exosome acting as a negative regulator of siRNA biogenesis. Our data indicate that the Arabidopsis exosome most likely lost this function during evolution, meaning that exosome substrates do not compete with siRNA precursors for siRNA biogenesis machinery and spurious transcripts do not enter RNAi pathways in plants. Additionally, it suggests that perhaps only very few of the ncRNA transcripts controlled by the exosome could be *bona fide* siRNA precursors. One of the reasons for this could be the fact that plants evolved two plant-specific RNA polymerases, Pol IV and Pol V, which specialize in siRNA-mediated TGS. Pol IV is required for biogenesis of the majority of 24-nt siRNAs and is supported by Pol V, which is responsible for production of a subset of siRNAs [31], [44], [45], [60]. It is also plausible that there might be other unknown plant-specific ribonucleases that specialize in controlling stability of siRNAs or the amount of siRNA precursors generated by Pol IV and/or Pol V in plants. We also cannot rule out the possibility that some of the transcripts controlled by the exosome in a small subset of loci are legitimate siRNA precursors; this definitely warrants further in-depth investigation.

### Exosome and DNA methylation-independent gene silencing

siRNA-dependent RdDM is thought to be the main pathway for transcriptional gene silencing of repetitive elements and transposons in plants [27], [28], [31], [61], [62], although existence of other DNA methylation-independent gene silencing pathways have also been reported [63]–[71]. One of the DNA methylation-independent gene silencing pathways is mediated by MOM1 (Morpheus' molecule 1) protein [63], [65], which predominantly silences transposons and loci harboring sequences related to *gypsy*-like transposons. Activation of transcription in *mom1* mutants occurs with no change in DNA methylation, histone modifications or chromatin condensation, and the investigation of the relationship between RdDM and MOM1 revealed a very complex interplay between these two pathways [63], [69], [72]–[74]. However, a reduction in H3K9 dimethylation was reported in some loci in *mom1* mutants and it was suggested that MOM1 may transduce RdDM signals to repressive histone modifications by an unknown mechanism [75].

Also, a recent study of MORC family ATPases revealed that mutation of *AtMORC1* or *AtMORC6* caused derepression of DNA methylated genes and TEs without any loss of DNA methylation, change in histone methylation or alteration of siRNA levels [71]. These proteins are involved in alteration of chromosome superstructure and are likely to act downstream of DNA methylation. These results indicate that there are multiple parallel pathways for DNA methylation-independent gene silencing in *Arabidopsis*. The exosome-mediated silencing we observed here bears some similarities to the silencing observed for MOM1 and MORC; for example, they show effects on repetitive sequences and an absence of effects on siRNAs, although there are notable differences as well. Here we show that, similar to MOM1 and MORC mechanisms, exosome-dependent gene silencing also affects repetitive sequences and acts independent of RdDM, although our results are limited in scope. Characterization of the relationship between these pathways remains an interesting topic for future study.

The different silencing pathways likely have different functions, depending on the genomic region, the nature of the regulated sequences, and the precision and dynamics of silencing required. For example, methylated sequences can affect the expression of nearby genes. The expression of nearby genes is negatively correlated with the density of methylated, but not unmethylated TEs. Methylated TEs are preferentially removed from gene-dense regions over time and TE families that have a higher proportion of methylated insertions are distributed farther from genes [76], arguing that positional effects and the surrounding landscape most likely contributes to the choice of silencing mechanisms and the interplay between them.

### How can exosome function in gene silencing in Arabidopsis?

There are multiple mechanisms by which the exosome can be envisioned to participate in gene silencing in Arabidopsis. Heterochromatin assembly is used by all eukaryotes in gene silencing. In addition to repressive histone modifications employed by all organisms, humans and plants widely use DNA methylation as well, and ncRNAs play a central role in the control of chromatin structure in all organisms. While ncRNA-mediated silencing proceeds through multiple mechanisms some of which are organism-specific, the end result appears to be the same repressive histone modifications. For example, budding yeast, which lacks RNAi machinery, employs strategies that include, but not limited to, the use of antisense, cryptic or read-through transcripts, as well as transcripts originating from divergent promoters to guide histone modifications. Fission yeast is more similar to higher eukaryotes and uses all of the above strategies in addition to utilizing RNAi as well. However, DNA methylation is not used by budding and fission yeast. Plants, on the other hand, evolved very sophisticated epigenetic mechanisms that include the use of both RNAi-dependent and RNAi-independent pathways to guide DNA methylation and histone modifications for gene silencing [9], [31]–[33], [44], [45], [47], [61], [68], [70], [75], [77], [78]. Exosome complex proved to be amazingly versatile in impacting gene silencing in budding and fission yeasts. In fission yeast, the organism which takes full advantage of RNAi machinery to regulate its gene expression, the exosome is involved in silencing of both facultative and constitutive heterochromatin by acting in several different pathways through smRNAs, produced in either an RNAi-dependent or RNAi-independent manner [38], [39], [79], [80]. It was also found to act through surveillance of RNA quantity and quality as well as by collaborating with termination machinery [40], [41], [57], [80], [81], similarly to the manner exosome participates in gene silencing in bakers yeast, which lacks RNAi machinery [55], [58], [59].

In Arabidopsis, silencing of repetitive elements involves siRNA-dependent DNA methylation guided by homologous siRNAs [9]. Repressive histone modifications always appear to accompany DNA methylation, however, the mechanistic link between them is not yet fully understood. In budding and fission yeasts, degradation of nuclear ncRNAs depends on polyadenylation by the TRAMP complex and involves Rrp6. We also found that mutations in two RRP6-like proteins AtRRP6 L1and AtRRP6 L2 led to significant dereperession of solo LTR (Figure 7B, 7C and 7D) and occurred in a DNA methylation-independent manner as in *rrp4* and *rrp41* (Figure 7F). These results suggest that Atrrp6s may be true nuclear catalytic subunits of Arabidopsis exosome, or may also work independently of core exosome. It will be interesting to examine whether another putative exosome catalytic subunit AtRrp44a [J. Lee and J. Chekanova unpublished data] is involved in this process, and whether components of the TRAMP complex also participate.

We also observed that the exosome physically associates with the polyadenylated ncRNA transcripts from scaffold producing regions. We could not reliably crosslink the exosome to the DNA of the target locus by ChIP (data not shown), although this could simply reflect the difficulty of reliably crosslinking proteins to DNA through RNA, or it could mean that the exosome binds to the transcripts after they are released from the locus and that exosome-mediated regulation of the transcripts may be important for maintenance of chromatin structure around the locus. H3K9 dimethylation was reported to be disturbed and lost when isolated Arabidopsis nuclei were treated with RNase A [82], meaning that histone modification may be affected by RNA level and/or RNA in close proximity to the target loci. In fission yeast, the mutation of *Cid14*, one of the subunits of the TRAMP complex, results in accumulation of aberrant heterochromatic RNA close to the target loci and leads to a mild decrease in H3K9 methylation. It was recently shown that decrease of H3K9 methylation in yeast is the result of HP1 protein (Heterochromatin Protein1), which binds to H3K9me2 heterochromatin and propagates H3K9me2 spreading, being titrated by an excess of heterochromatic RNA [83]. In our study, we also observed a combination of the transcripts accumulation in exosome mutants relative to WT with a weak decrease in H3K9me2 levels in solo LTR (Figure 6A). Taken together, these data could suggest that a similar mechanism to regulate the stability of chromatin structure might operate in plants. However, LHP1 (Like-HP1), the closest Arabidopsis homolog of yeast HP1, has specificity for H3K27me3 [84], not H3K9me2, and the *rrp41 iRNAi/nrpe1* double mutant did not exhibit any additive or synergistic effect on the loss of H3K9me2 relative to respective single mutants as well, suggesting that the loss of H3K9me2 observed in the exosome mutants is unlikely to result from an unknown functional homolog of *Arabidopsis* HP1 simply titrating an excess of ncRNA off chromatin, as reported in fission yeast.

**Figure 8:**
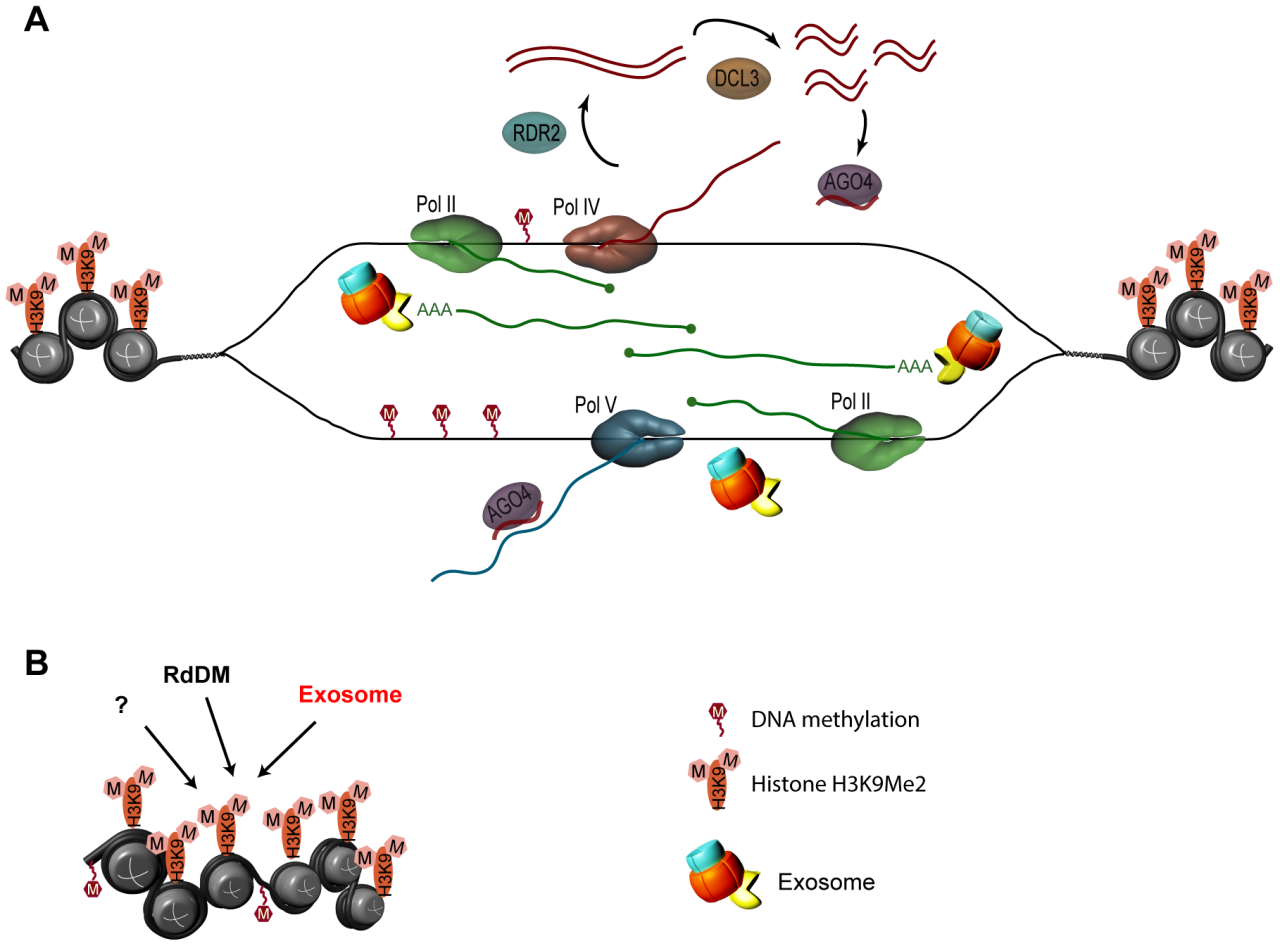
Model for the role of the exosome complex in gene silencing at solo LTR in *Arabidopsis*. The process of silencing of the solo LTR locus is substantially more complex than portrayed here, but for simplicity only the factors examined in this study are represented. No modifications to the prevailing views on the roles of Pol IV, Pol V, Pol II, RDR2, DCL3, and AGO4 are proposed [9], [28], [30], [31], [60]. A. The exosome complex is not involved in the regulation of quality or quantity of siRNAs produced from region A. RNA Pol II (green) generates transcripts from region B of solo LTR. It is also possible that Pol II transcribes both A and B regions in opposite directions. Either Pol II transcripts or the process of transcription from region B recruits Pol V (blue), complexed with AGO4 and siRNA, to the scaffold-producing region B. Due to the sequence complementarity between siRNAs, which are derived from region A only, and the portion of the scaffold transcripts that partially overlaps with region A, AGO4/siRNA RISC localizes to region A and recruits other components of the silencing machinery. Both Pol II and Pol V were implicated in producing region B scaffold transcripts [30], [31]. Exosome is not involved in siRNA metabolism and does not contribute to DNA methylation. Exosome participates in controlling the amount of top transcripts emanating from the scaffold-producing region B of solo LTR, and thus may contribute to the repression of region A through regulating the level of region B transcripts. The exosome associates with transcripts emanating from the scaffold-producing region and plays a role in locus silencing through maintaining or establishing chromatin structure. B. More than one silencing pathway controls the solo LTR locus. The exosome associates with transcripts emanating from the adjacent scaffold-producing region, and plays a role in locus silencing through maintaining or establishing chromatin structure by affecting histone methylation (H3K9), in parallel to the RdDM pathway, which affects siRNAs and DNA methylation (“M” in red hexagons).

Our results showed that the exosome depletion produced no effect on siRNAs and DNA methylation of solo LTR, AtSN1 and IGN5 loci, arguing that the exosome complex functions independently of RdDM. However, our findings also indicated that the exosome is involved in the silencing of these loci and does interact with the RdDM pathway, possibly through its functional interaction with RNA Pol V. The converging transcripts we observed in the *rrp41-i* and *rrp4-i* mutants in solo LTR and AtSN1 suggest that the exosome is involved in regulation of either processing or level of RNA from these loci (Figure 4A–4I, and model Figure 8). We found that production of smRNAs from the siRNA-generating A regions was totally abolished in *rrp41/nrpd1* double mutant (Figure 5A–5D), ruling out a possibility for these transcripts to serve as a double stranded precursors for RNA Pol IV-independent siRNAs. We also found that the exosome physically associates with the polyadenylated transcripts produced from the scaffold region (region B) and exhibits synergistic derepression of the locus (region A) when combined with a Pol V mutant, while there was no change in the derepression in *rrp41/nrpd1* double mutants (Figure 4B, 4C, 4H and 4I). Based on these results, we speculate that RNA polymerase V may function in gene silencing of these loci in two ways, the first acting in the DNA- methylation-dependent RdDM pathway, and the second acting independently of a DNA-methylation. Indeed, RdDM- independent roles of Pol V in silencing of 5S rDNA [31], [85] and several other loci [82] were previously reported. A recent genome-wide study of Pol V-associated loci also hints at the possibility of Pol V having unknown functions in addition to the function it plays in the RdDM pathway [45]. The DNA-methylation-independent function of Pol V may then be in addition to its function in RdDM, and may operate in parallel to the exosome pathway. If this is the case, the depletion of both *rrp41* and *nrpd1* may not lead to synergistic derepression because it would be compensated by the RdDM-independent function of Pol V. However, deficiencies in exosome and Pol V would result in synergistic desilencing due to the loss of three different pathways. Both Pol II and Pol V were reported to be responsible for the transcription of scaffold RNA and be required for silencing [30], [31], although it is not known how their activities are functionally integrated. It is also not known how Pol V initiation sites are chosen, but they appear to be promoter independent [31]. Perhaps transcription by Pol II helps maintain open chromatin architecture at this site, and together with the resulting noncoding RNAs facilitates Pol V transcription initiation. Alternative possibility is that Pol II produces two distinct pools of transcripts, one of which is controlled by the exosome, and the exosome functions by regulating the Pol II transcripts that are distinct from the transcripts that are used in RdDM pathway. This possibility would be very interesting to examine, particularly in light of the yeast exosome involvement in gene silencing through regulation of cryptic transcripts, transcripts originating from divergent promoters and read-through transcripts [4], [55], [58], [59]. How the Arabidopsis exosome complex and the exosome controlled ncRNAs facilitate recruitment of chromatin modifiers in order to enforce silencing through repressive histone modifications remains an interesting topic of future studies. We suggest that the exosome may coordinate the transcriptional interplay of RNA polymerases Pol II and Pol V to achieve the right level of transcriptional repression of heterochromatic loci (Figure 8).

In summary, our data suggest that the exosome likely acts in a parallel pathway to RdDM pathways in gene silencing, possibly affecting the transcriptional interplay of different RNA polymerases to modulate repression of heterochromatic sequences. The mechanisms that link this RNA metabolic complex, the epigenetic modification of histone methylation, and heterochromatic silencing in plants remain to be elucidated. Our results indicate that there is no one-size-fits-all pathway or mechanism that exclusively governs silencing of all loci; rather, different loci and different players in RdDM interact with different pathways and are silenced by different, likely overlapping mechanisms. The positional effects and the surrounding landscape most likely also play important roles in the choice of silencing mechanisms and the interplay between them. This may reflect the crucial importance of silencing in developmental gene regulation and in maintenance of genomic stability by suppression of invasive sequences.

## Materials and Methods

### Plant materials and growth conditions

iRNAi lines of exosome subunits RRP4 and RRP41, RNA Pol IV (SALK_128428.20.10, *nrpd1a-3*, *nrpd1-3*), RNA Pol V (SALK_029919, *nrpd1b-11*, nrpe1-11), RDR2 ( SAIL_1277808, *rdr2-1*), and DCL3 ( SALK_005512.38.70.x0, *dcl3-1*) mutants were described previously [1], [27], [33], [86]. *rrp41 iRNAi/nrpd1-3, rrp41 iRNAi/nrpe1-11, rrp4 iRNAi/nrpd1-3*,and *rrp4 iRNAi/nrpe1-11* double mutants were obtained by crossing of *rrp41 iRNAi* and *rrp4 iRNAi* with *nrpd1*/*nrpe1-11* line. *rrp41 iRNAi/dcl3-1, rrp41 iRNAi/rdr2-1* double mutants were obtained by crossing.

The alleles of the *rrp6l2-2* and *rrp6l3-1* correspond to SALK_011429 and SALK_122492 lines, respectively. The *rrp6l1-2* allele was isolated from the University of Wisconsin BASTA population. The ecotype background is Col-0 for all Salk alleles and Ws for University of Wisconsin alleles. To induce *iRNAi*, seedlings were germinated and grown for 7 days on ½× MS plates with 8 mM 17β-estradiol, as described before [1].

### Library construction

Total RNA was isolated from 7-day-old seedlings using the mirVana miRNA isolation kit (Ambion) according to the manufacturer's protocol. The total RNA sample was used for sequencing library construction using the Small RNA sample Prep v1.5 kit and TruSeq Small RNA Sample Prep kit (Illumina, San Diego, CA) according to the manufacturer's instructions. The smRNA libraries were sequenced using the Illumina Genetic Analyzer II (by DNA Core Facility, University of Missouri) and Illumina HiSeq 2000 (by Biotechnology Center, University of Wisconsin) according to the manufacturer's instructions. HiSeq 2000 sequencing reads were demultiplexed using Casava v 1.8 (by Bioinformatic Resource Center, University of Wisconsin) before further bioinformatic analysis

### Bioinformatic analysis of small RNAs

Data processing was done using available tools and custom in-house UNIX shell programming [43], [75], [87]–[90]. The raw sequences in Illumina GAIIx and demultiplexed HiSeq 2000 sequencing reads were trimmed removing adapter using “fastx_clipper” in the FASTX-Toolkit (version 0.0.13) [91] and smRNAs with lengths between 15- and 32-nt were selected and mapped to the Arabidopsis genomic sequences (TAIR9 version) using BOWTIE (version 0.12.7) [92]. Reads that failed to perfectly map to the nuclear genome with no mismatches, and reads present in fewer than two counts were discarded. All *Arabidopsis* lines used in this study carried *iRNAi* cassette transgenes used for inactivation of either RRP4 or RRP41 exosome subunit genes [1]. These silencing cassettes generate a number of 21-, 22- and 24-nt silencer sequences corresponding to RRP4 or RRP41 genes (mapping to AT1G03360 and AT3G61620 loci), respectively. Therefore, silencer sequences produced from *iRNAi* transgenes were filtered out from each library and libraries were analyzed separately to ensure accurate interpretations. The remaining smRNA reads, termed FLR for filtered reads, were used for further analysis.

Each library was normalized either to the total number of mapped non-redundant reads or to the total number of non-redundant filtered reads (FLR), multiplied by 10^6^ (rpm, reads per million). Both methods of normalizations were compared and found to produce results which lead to identical interpretations, therefore, only data analyzed using filtered reads are presented in this study.

Classification of small RNAs was performed by BEDTools (v2.10.0) [93] and in-house UNIX shell programming using the following databases: TAIR9 annotations for protein coding and non-coding features (tRNA, rRNA, ncNRA, miRNA, snRNA, snoRNA, and transposable elements [76]), miRBase (release 18) [94] or mature miRNA annotations. Some smRNAs match more than one annotation category; therefore the sum of the numbers is bigger than the total input number.

The small RNA reads with 20 to 25 nt length were calculated and plotted versus the sum of their normalized reads per million (rpm). The relative frequencies of each 5′ terminal nucleotide of the small RNAs were calculated (Tables S1, S2 ) and represented graphically.

Repetitive genomic features were classified using TAIR9 Tandem Repeat Finder (version 4.04) [95] and Inverted Repeat Finder (version 3.05) [96]. Annotation of dispersed repeats was done with Repeat Masker (version 3-3-0) [97].

For analysis of locus-specific expression of smRNAs (solo LTR, AtSN1, IGN5, REG3, and REG4), the expressed normalized reads per million (rpm) were calculated for respective genomic locus and locus-specific datasets were plotted for comparisons.

### RNA analysis

Total RNA was isolated from 7-day-old seedlings using the Trizol method. For RT-qPCR, 1–4 µg of total RNA digested with DNase I (Fermentas) was reverse transcribed 1 hour either at 50°C (for oligo-dT primer) or 55°C (for specific primers) using 60–100 units SuperScript III Reverse Transcriptase (Invitrogen). Transcripts were quantified by RT-qPCR using the comparative threshold cycle method (ΔΔC_t_, primers listed in Table S4), using *Actin2* (At3g18780) as endogenous reference. Polyacrylamide Northern Blot analyses were performed as described [25].

### Analysis of DNA methylation

Genomic DNA was isolated from 7-day-old seedlings using a DNeasy kit (QIAGEN). The methylation analysis using DNA sensitive methylation enzymes was followed as described [27], [31], [77].

### Chromatin immunoprecipitation (ChIP) assays

ChIP was performed as described [98]. One gram of 7-day-old seedlings was used for each experiment. All ChIP experiments were reproduced at least twice on each of the two or more biological replicates. The normalization was done relative to input using [99]. Anti-RNA Pol II (ab817) and anti-H3K9me2 (ab1220) were obtained from Abcam, and anti-H3K27me1 antibody from Upstate. An equal amount of chromatin not treated with antibody was used as the mock antibody control. The ChIPed DNA was purified using PCR purification kit (Fermentas) before being used for qPCR. The primer sets used for the PCR are listed in Table S4.

### RNA immunoprecipitation (RIP)

RIP assays were performed by adapting an existing protocol [100]. Transgenic plants expressing TAP-tagged RRP41 at physiological levels [1] were used in the experiment. Two grams of 2-week-old seedlings were collected and fixed with 1% formaldehyde. For RRP41-RNA complex purification, the chromatin solution was incubated overnight with prewashed IgG Sepharose 6 Fast Flow (GE Healthcare) at 4°C. Immunoprecipitated RNA was purified with phenol: chloroform and cDNA synthesis was performed using SuperScript III reverse transcriptase (Invitrogen) and random hexamers (Promega). The primer sets used for the PCR are listed in Table S4.

## Supporting Information

Figure S1 *iRNAi* silencer sequences produced by *rrp4-i* and *rrp41-i* cassettes in response to estradiol treatment. (A, B) 20–25 nt smRNAs corresponding RRP4 in *rrp4-i* (A) and corresponding and to RRP4 in *rrp41-i* (B) depletion mutants profiled based on the length of the reads. (C, D) 20–25 nt smRNAs produced from in *rrp4-i* (C) and *rrp4-i* (D) depletion mutants profiled based on both their length and the terminal 5′ nucleotide. The major silencer sequences are 5′U and 5′A smRNA species.
(TIF)

Figure S2 miRNA families, miR-158a, miR-158b, miR-860, miR-823, miR-841, miR-5561 and variations in sequence length. miRNA families miR-158a, miR-158b, miR-860, miR-823, miR-841, and miR-5561 and variations in sequence length in each family. smRNAs mapped to matching mature miR-158, miR-860, miR-823, miR-841, and miR-5561 sequences [94](miRBase release 18) were plotted versus the sum of their normalized reads per million (rpm) from smRNA libraries constructed from RRP4, *rrp4-i*, RRP41, *rrp41-i*, RRP41*/nrpd1, rrp41 iRNAi/nrpd1*, RRP4 *iRNAi/nrpe1 and rrp41 iRNAi/nrpd1* mutants.
(TIF)

Figure S3 Effects of exosome deletion, RdDM, and other mutants. (A) Diagrams of IGN5, REG 3 and REG 4 genomic loci, based on analysis of transcription units by Wierzbicki et al. (2008) [1], [31]. Region A corresponds to siRNA producing region, region B corresponds to scaffold producing region in both loci, red lines mark regions amplified in RT-PCR and qPCR. (B) 20–25 nt smRNAs produced from region A of IGN5 in *rrp4-i, rrp41-i* exosome depletion lines and RdDM mutants. All locus-specific datasets of 20–25 nt smRNAs are plotted versus the sum of their normalized reads per million (rpm). (C, D) RT-PCR analysis of RRP6L1 and RRP6L mRNA expression in *rrp6L1* and *rrp6L2* insertion mutants.
(TIF)

Table S1 Summary of smRNA sequence reads in the libraries of RRP4, rr*p4-i*, RRP41, and *rrp41-i* plants.
(XLS)

Table S2 Summary of smRNA sequence reads in the libraries of RRP41/*nrpd1, rrp41-i/nrpd1*, RRP41/*nrpe1*, *rrp41-i/nrpe1*, RRP41/*rdr2, rrp41-i/rdr2*, RRP41/*dcl3* and *rrp41-i/dcl3* plants.
(XLS)

Table S3 Expression profiling of known mature miRNAs in the libraries of RRP4, rr*p4-i*, RRP41, and *rrp41-i* mutant plants.
(XLS)

Table S4 Oligonucleotides used in this study.
(XLS)
