# Supplementary figures and images for: The Role of the Arabidopsis Exosome in siRNA–Independent Silencing of Heterochromatic Loci
Source: PLoS Genet. 2013 Mar 28;9(3):e1003411. doi: 10.1371/journal.pgen.1003411 (PMC3610620; doi:10.1371/journal.pgen.1003411)

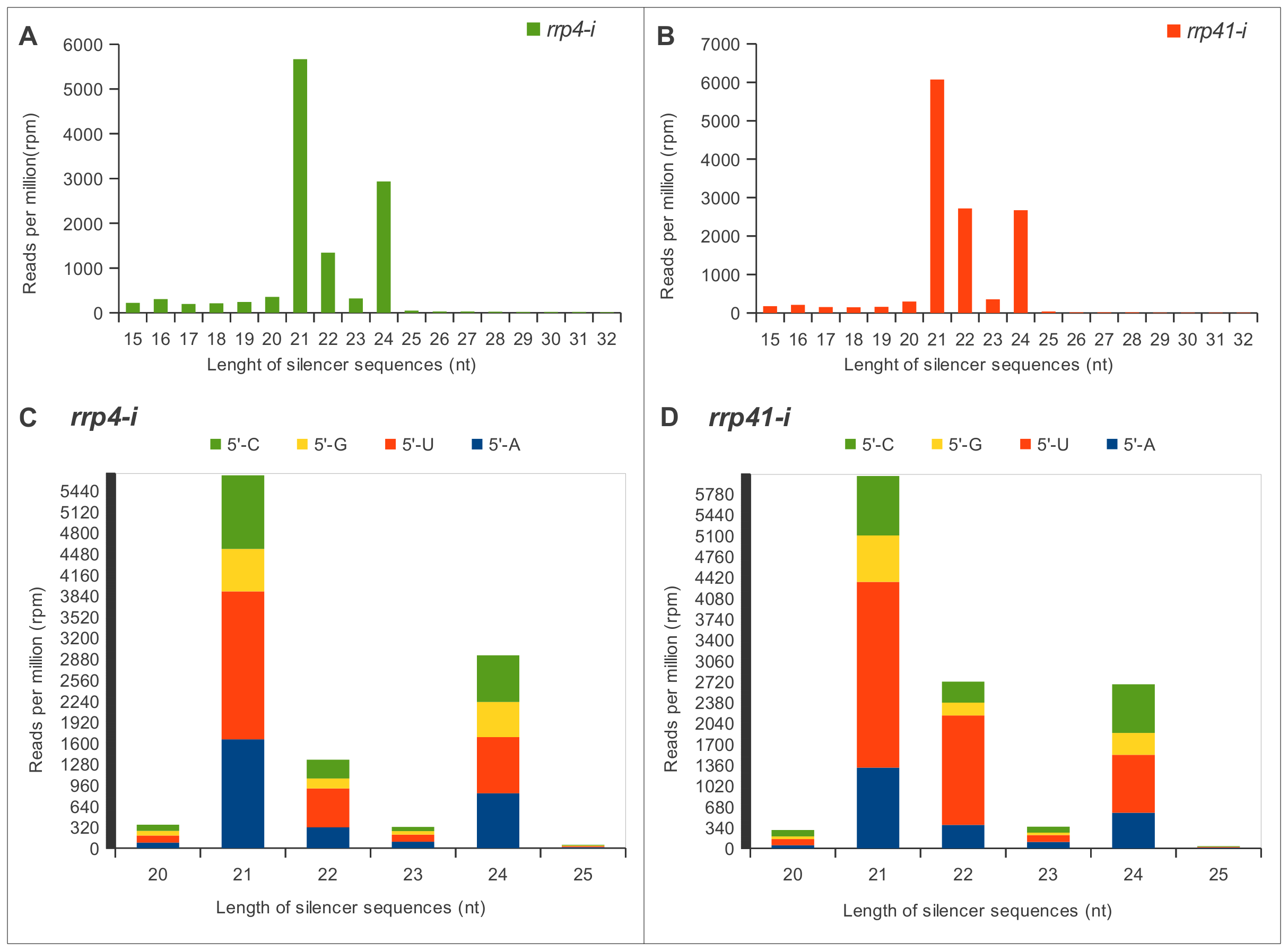

Supplement: Figure S1 — iRNAi silencer sequences produced by rrp4-i and rrp41-i cassettes in response to estradiol treatment. (A, B) 20–25 nt smRNAs corresponding RRP4 in rrp4-i (A) and corresponding and to RRP4 in rrp41-i (B) depletion mutants profiled based on the length of the reads. (C, D) 20–25 nt smRNAs produced from in rrp4-i (C) and rrp4-i (D) depletion mutants profiled based on both their length and the terminal 5′ nucleotide. The major silencer sequences are 5′U and 5′A smRNA species. (TIF) [file pgen.1003411.s001.tif]

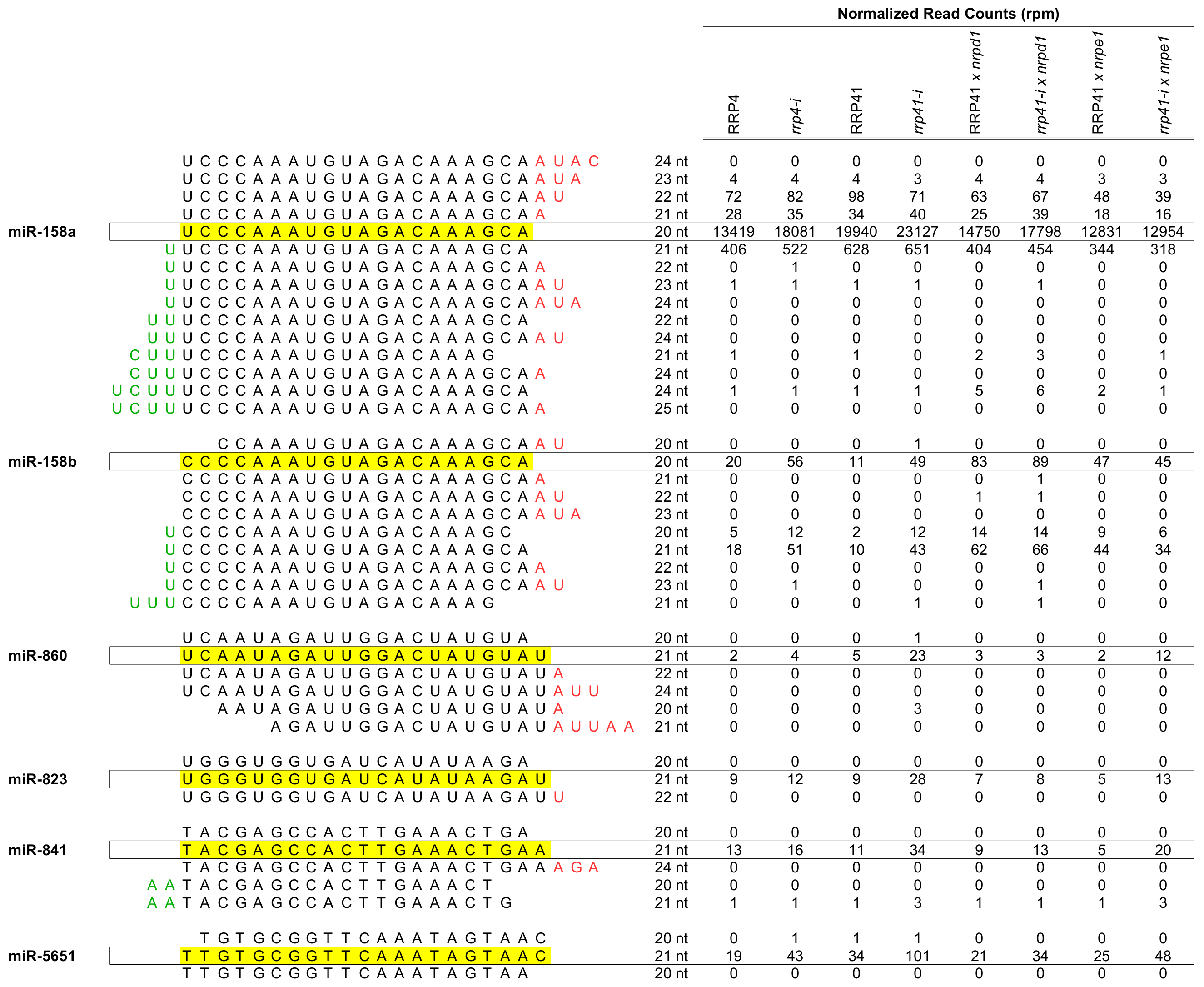

Supplement: Figure S2 — miRNA families, miR-158a, miR-158b, miR-860, miR-823, miR-841, miR-5561 and variations in sequence length. miRNA families miR-158a, miR-158b, miR-860, miR-823, miR-841, and miR-5561 and variations in sequence length in each family. smRNAs mapped to matching mature miR-158, miR-860, miR-823, miR-841, and miR-5561 sequences [94](miRBase release 18) were plotted versus the sum of their normalized reads per million (rpm) from smRNA libraries constructed from RRP4, rrp4-i, RRP41, rrp41-i, RRP41/nrpd1, rrp41 iRNAi/nrpd1, RRP4 iRNAi/nrpe1 and rrp41 iRNAi/nrpd1 mutants. (TIF) [file pgen.1003411.s002.tif]

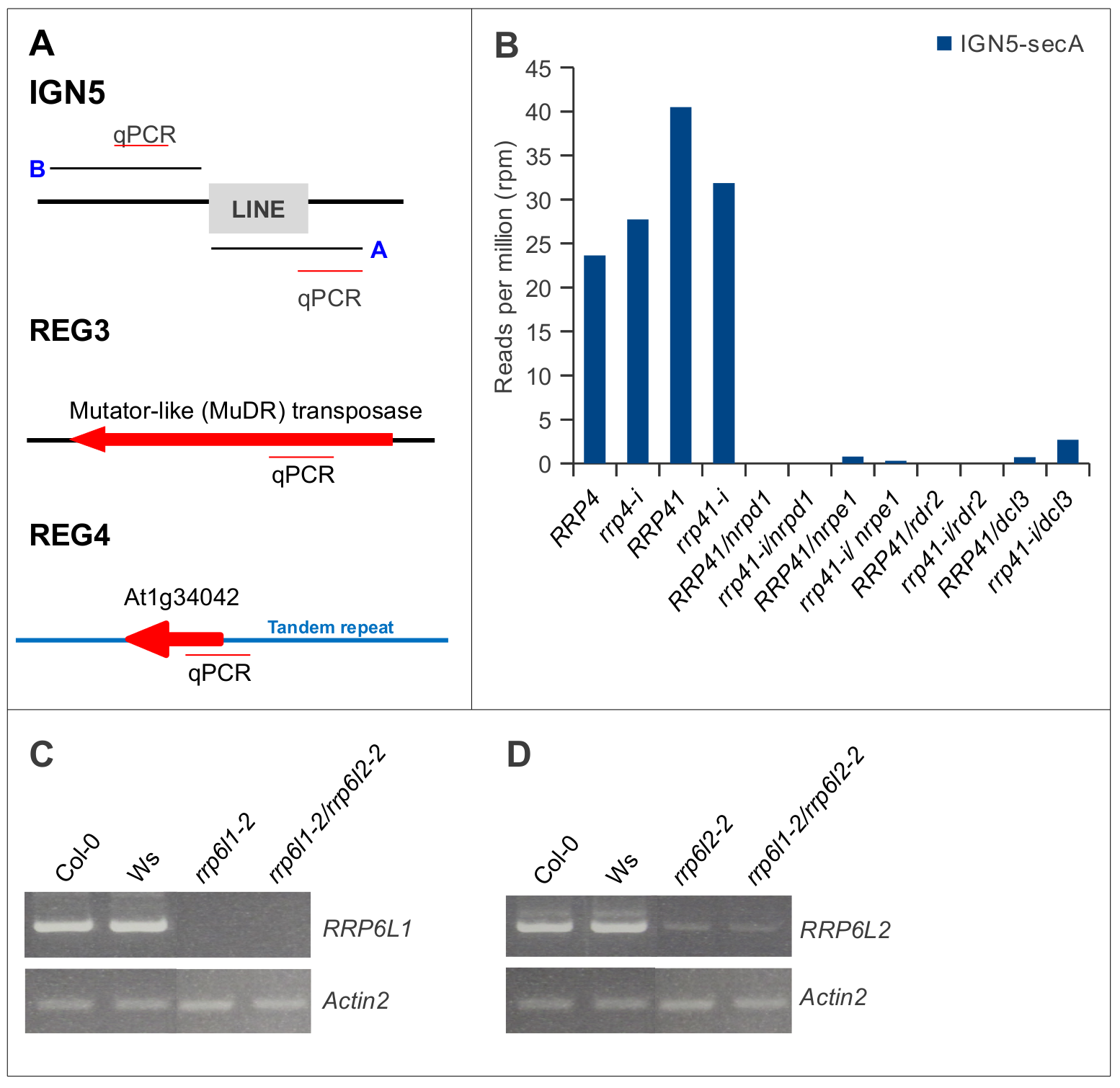

Supplement: Figure S3 — Effects of exosome deletion, RdDM, and other mutants. (A) Diagrams of IGN5, REG 3 and REG 4 genomic loci, based on analysis of transcription units by Wierzbicki et al. (2008) [1], [31]. Region A corresponds to siRNA producing region, region B corresponds to scaffold producing region in both loci, red lines mark regions amplified in RT-PCR and qPCR. (B) 20–25 nt smRNAs produced from region A of IGN5 in rrp4-i, rrp41-i exosome depletion lines and RdDM mutants. All locus-specific datasets of 20–25 nt smRNAs are plotted versus the sum of their normalized reads per million (rpm). (C, D) RT-PCR analysis of RRP6L1 and RRP6L mRNA expression in rrp6L1 and rrp6L2 insertion mutants. (TIF) [file pgen.1003411.s003.tif]
